# Battery Fluid

**Published:** 1879-09

**Authors:** 


### BATTERY FLUID.

Dr. Tilley, in the Chicago *Medical Journal and Examiner*, makes public a device by which the constancy of the galvanic current may be increased and the necessity of frequently re-amalgamating the zincs avoided. The idea is not original with him, but it is not generally known, and any device which will dispense with the necessity of amalgamating the zincs, will be a great source of satisfaction to the majority of physicians. To accomplish this it is necessary only to add a certain quantity of bisulphate of mercury to the ordinary battery fluid. The amount given by Gaiffe, of Paris, is four or five grammes of the bisulphate of mercury to the liter of the regular sulphuric acid and bichromate fluid. Of course it is necessary to amalgamate the zincs before commencing its use. They will then remain amalgamated. The marked improvement in the constancy of the current obtained, and the avoidance of the troublesome process of reamalgamating the zincs, makes this hint a valuable one to all physicians using batteries.

